# Hepatitis B Surface Antigen Loss and Improved Clinical Outcomes in Asians with Chronic Hepatitis B Virus Infection

**DOI:** 10.1016/j.gastha.2025.100844

**Published:** 2025-11-06

**Authors:** Wallis Lau, Myriam Drysdale, Eleonora Morais, Luis Antunes, Loey Mak, Christopher Lee, Catarina Camarinha, Xiaohui Sun, Adrienne Y.L. Chan, May Lam, Vera Gielen, Dickens Theodore, Ian C.K. Wong, Iain A. Gillespie

**Affiliations:** 1 Department of Pharmacology and Pharmacy, The University of Hong Kong, Hong Kong, China; 2 UCL School of Pharmacy, London, UK; 3 Laboratory of Data Discovery for Health (D24H), Hong Kong Science and Technology Park, Hong Kong, China; 4 GSK, London, UK; 5 IQVIA, Lisbon, Portugal; 6 Department of Medicine, The University of Hong Kong, Hong Kong, China; 7 IQVIA, London, UK; 8 Aston Pharmacy School, Aston University, Birmingham, UK; 9 GSK, Durham, North Carolina; 10 GSK, Stevenage, UK

**Keywords:** Asian, Clinical Outcomes, HBsAg Loss

## Abstract

**Background and aims:**

Chronic hepatitis B virus (HBV) infection accounts for substantial disease burden and mortality due to liver complications. Hepatitis B surface antigen (HBsAg) loss is a key component of functional cure when assessing treatment efficacy. However, the impact of HBsAg loss on clinical outcomes deserves further exploration.

**Methods:**

This population-based cohort study used electronic health record data from a territory-wide database in Hong Kong to identify patients with chronic HBV infection (2005–2019). The association between HBsAg loss and outcomes was assessed: compensated cirrhosis, decompensated liver disease (DLD), hepatocellular carcinoma (HCC), and all-cause mortality (ACM). A marginal structural model using inverse probability weighting was used to estimate hazard ratios (HRs; 95% confidence interval [CI]) adjusted for time-fixed and time-varying confounders. Health-care resource utilization before and after loss was evaluated.

**Results:**

The study population comprised 71,077 patients accruing 348,379 person-years; 1639 (2.3%) experienced HBsAg loss, which occurred with a mean (standard deviation) of 74.63 (37.5) months after chronic HBV index date. HBsAg loss was associated with a reduced risk of DLD (74%; HR 0.26 [95% CI 0.08–0.83]), HCC (66%; 0.34 [0.19–0.61]), and ACM (26%; 0.74 [0.57–0.97]). The HR for compensated cirrhosis was 0.57 (0.30–1.14). Each additional month of HBsAg loss was associated with decreased risk of HCC and ACM. Of those experiencing HBsAg loss, cumulative probability of persistence at 24 and 60 months was 99% and 97%, respectively. Hospital admission, inpatient days, and drug prescribing were higher before HBsAg loss versus 6, 12, and 24 months post-HBsAg loss.

**Conclusion:**

In this large population-based study with extended follow-up in Hong Kong, HBsAg loss was associated with reduced risk of DLD, HCC, and ACM.

The burden of chronic hepatitis B virus (cHBV) infection in the Asia-Pacific region is high, accounting for 65% of the estimated 254 million cases worldwide.^1^^,^^2^ In 2022, the estimated prevalence of cHBV infection in those aged 15–84 years in Hong Kong was 6.2%.^3^ Vaccines effective against hepatitis B virus (HBV) have been available in Hong Kong since 1982; however, prevalence remains high among those born before the HBV vaccine became available, and population-level reductions in HBV carriage have occurred only very gradually.^4^ In 2019, 357,000 deaths in the Asia-Pacific region were attributable to HBV infection.^2^

Current treatments for cHBV infection, which include interferon-based drugs and nucleot(s)ide analogs (NAs), aim to achieve continuous viral suppression and prevent disease progression. Interferon therapy is typically administered up to 48 weeks, with numerous contraindications and poor tolerance, whereas NA therapy can be indefinite.^5^

Presence of hepatitis B surface antigen (HBsAg) is a hallmark of HBV infection, and contributes to exhausted T-cell immunity and failure to clear infection.^6^^,^^7^ Chronic HBV infection is indicated by HBsAg presence for ≥6 months.^6^ HBsAg seroclearance is infrequent, both naturally and with current treatment options.^8^ The yearly incidence of spontaneous seroclearance is ∼1%, and seroclearance rates with current standard-of-care treatment options such as NAs and interferon range from 1%–7%.^8^^,^^9^ Although current treatments mainly target suppression of HBV replication, the aim of new drugs in development is functional cure, defined as sustained HBsAg loss and HBV DNA below the lower limit of quantification 24 weeks after discontinuation of cHBV treatment.^10^ Functional cure is now regarded as an optimal endpoint for HBV treatment,^11^ with HBsAg loss being used as a proxy of functional cure in observational studies,^10^ as contemporaneous presence of HBsAg and HBV DNA is frequently lacking in existing databases.

The association between HBsAg loss and improved long-term clinical outcomes warrants further exploration. Much variability is observed across studies, particularly in terms of population, design outcomes, and exposure definitions. The accurate quantification of these associations is often limited by short follow-up periods, low event numbers, and population heterogeneity.^8^^,^^12^^,^^13^ In this large population-based study with extended follow-up, we investigated the association between HBsAg loss and clinical outcomes in a cohort of adults from Hong Kong with cHBV infection. This study is part of an international collaboration, with similar studies being conducted in the US and Europe.

## Methods

### Study Design and Data Source

**Figure 1:**
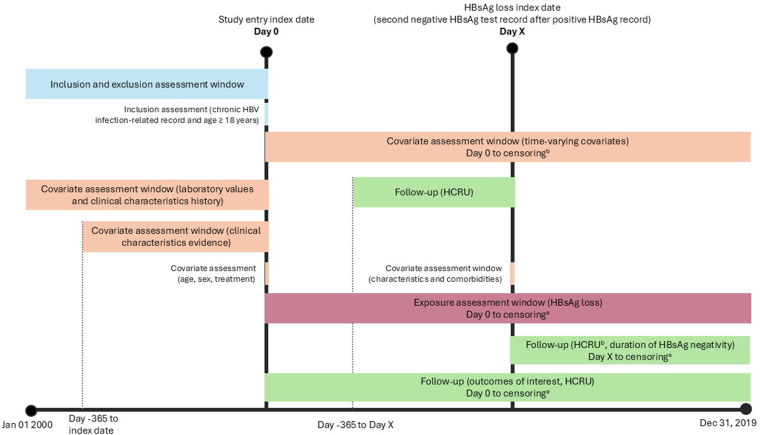
Study design. (a) Earliest of either end of the study period or death. (b) For patients who experienced HBsAg loss, HCRU after HBsAg loss was described both between HBsAg loss index and end of follow-up and for the first 6 months, 1 year, 2 years, and 5 years after HBsAg loss index.

This study used data from January 1, 2000, to December 31, 2019, with a cohort identification period from January 1, 2005, to December 31, 2019 (Figure 1), and used routinely collected electronic health record (EHR) data from the Clinical Data Analysis and Reporting System (CDARS).^14^ CDARS is a territory-wide database of the Hong Kong Hospital Authority, a statutory body managing all public hospitals and their ambulatory (general and specialist) clinics in Hong Kong. The database contains deidentified patient-level data from linked EHRs, including demographics, prescriptions, pharmacy dispensing, diagnosis (International Classification of Diseases, Ninth Revision), laboratory test results, procedures, admission, and discharge information. This study was approved by the Institutional Review Board of the University of Hong Kong/Hospital Authority Hong Kong West Cluster (Ref: UW 18–471). No direct subject contact or primary collection of individual human subject data occurred.

### Study Population

Patients (aged ≥18 years) with a diagnosis of cHBV infection between January 1, 2005, to December 31, 2019, were identified in CDARS based on either having (i) at least one cHBV infection diagnostic code or (ii) 2 positive serum HBsAg test results ≥6 months apart. The cHBV index date was the date of first evidence of cHBV infection. The study entry index date was the date when both HBV DNA and alanine aminotransferase (ALT) had been first recorded on or post-cHBV index date. HBsAg loss was defined as the first instance of ≥2 consecutive negative laboratory results, ≥6 months apart, with the second test forming the loss index date (Table A1).

Patients were excluded if they had ≥1 negative HBsAg laboratory result, received immunosuppressants, or had coinfection with human immunodeficiency virus (ever), hepatitis C virus, or hepatitis D virus (prior to or at study entry).

### Study Outcomes

The primary study outcomes were the incidence of compensated cirrhosis (CC), decompensated liver disease (DLD), hepatocellular carcinoma (HCC), and all-cause mortality (ACM) (detailed in Appendix). Patients with history of the outcome at baseline were excluded from the analysis assessing the relationship between that outcome and HBsAg loss. Patients were followed until the occurrence of an outcome, death, or end of the study period, whichever came first. Two approaches, “as-treated” (patients censored on seroreversion, defined as ≥2 consecutive positive HBsAg results after loss, the latter being considered the seroreversion date) and “intention-to-treat” (patients having HBsAg loss were not censored on seroreversion), were explored. Secondary outcomes included durability of HBsAg loss and health-care resource utilization (HCRU; all-cause and liver-related hospital admissions, days of hospitalization, NA treatment, all treatment).

### Statistical Analysis

#### Primary outcomes

The association between HBsAg loss and outcomes was investigated through marginal structural modeling (MSM) and inverse probability weighting (IPW) to account for both fixed and time-varying confounders (eg, anti-HBV treatment, ALT, HBV DNA). We estimated time-varying IPWs for each month by fitting a pooled logistic regression model for the monthly probabilities of HBsAg loss (exposure) and for remaining uncensored due to death. HBsAg loss was defined as a binary variable (loss or no loss) and as a quantitative variable (the effect of each additional month of HBsAg loss).

In the as-treated analysis, additional IPWs were derived for the monthly probability of remaining uncensored due to seroreversion. The effects of HBsAg loss on each outcome were estimated using a weighted pooled logistic regression model. The odd ratios generated approximated to hazard ratios (HRs) from a Cox model.^15^ Details of variables are listed in Table A2.

Subgroup analyses were conducted in NA-treated patients and by baseline cirrhosis status at study entry. NA-treated patients were defined as having no changes to their NA regimen for ≥6 continuous months at baseline. Only 267 (1.78%) of the 15,033 NA-treated patients experienced HBsAg loss; the results of this underpowered analysis are described in the Appendix.

#### Secondary Outcomes

Kaplan–Meier analysis was performed on the HBsAg loss group, considering time from loss index date to the date of seroreversion, and stratified by sex, hepatitis B e antigen (HBeAg) status, treatment and cirrhosis history. HCRU was calculated cumulatively until the end of follow-up. In patients who experienced HBsAg loss, HCRU was stratified by periods of time relative to the occurrence of loss (12 months pre-loss; 6 months, 1, 2, and 5 years post-loss index date).

## Results

### Patient Characteristics–Demographics

**Figure 2:**
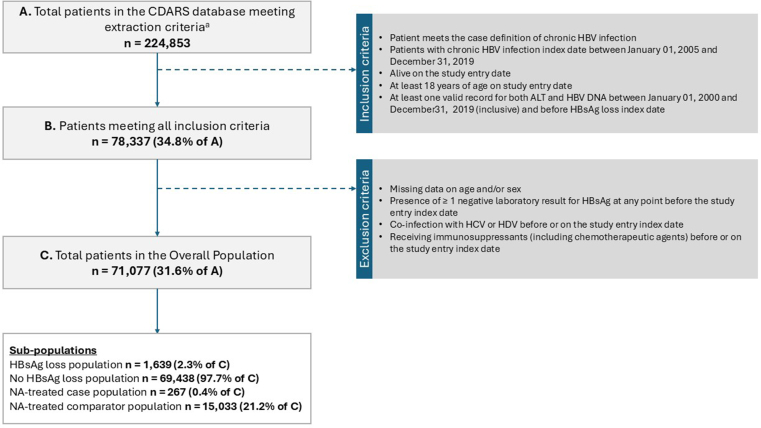
Study population. (a) Patients who received at least 1 diagnosis of acute HBV infection, at least 1 diagnosis of chronic HBV infection, or at least 1 positive HBsAg test result during the study period from January 1, 2000, to December 31, 2019, with no diagnosis of human immunodeficiency virus at any time before or on December 31, 2019. HCV, hepatitis C virus; HDV, hepatitis D virus.

The study population comprised 71,077 patients accruing 348,379 person-years (Figure 2). Of these, 1639 patients (2.3%) experienced HBsAg loss during follow-up (“loss patients”) and 69,438 patients (97.7%) did not (“no-loss patients”); where observed, HBsAg loss occurred with a mean (standard deviation [SD]) of 74.63 (37.5) months after study entry. In the overall population, the mean age at study entry was 52.7 years (SD 13.7), 41,238 (57.8%) were males, and 65.8% were untreated (Table 1).

**Table 1:** Selected Baseline Characteristics

	HBsAg loss N = 1639		No HBsAg loss N = 69,438		Overall population N = 71,077	
	n	% (95% CI)	n	% (95% CI)	n	% (95% CI)
Demographics at study entry index date						
Age (continuous, in y)						
N (%)	1639 (100)		69,438 (100)		71,077 (100)	
Mean (SD)	52.51 (11.07)		52.74 (13.73)		52.73 (13.67)	
Median (Q1–Q3)	53 (46–60)		53 (43–62)		53 (43–62)	
Min–Max	20–83		18–103		18-103	
Sex						
Female	533	32.5 (30.3–34.8)	29,306	42.2 (41.8–42.6)	29,839	42.0 (41.6–42.3)
Male	1106	67.5 (65.2–69.7)	40,132	57.8 (57.4–58.2)	41,238	58.0 (57.7–58.4)
Time since chronic HBV infection index date						
Time since index date (in mo) at study entry index date						
N	1639 (100)		69,438 (100)		71,077 (100)	
Mean (SD)	23.92 (27.75)		21.88 (32.48)		21.93 (32.38)	
Median (Q1–Q3)	15.03 (0–38.29)		2.99 (0–34.21)		3.22 (0–34.31)	
Min–Max	0–143.85		0–176.55		0–176.55	
Time since index date (in mo) at loss index date (HBsAg loss group only)						
N	1639 (100)		NA	NA	NA	NA
Mean (SD)	74.63 (37.52)		NA	NA	NA	NA
Median (Q1–Q3)	72.37 (45.36–101.74)		NA	NA	NA	NA
Min–Max	6.22–177.93		NA	NA	NA	NA
Clinical history at the study entry index date						
Liver fibrosis and cirrhosis						
No evidence/history of liver fibrosis or cirrhosis	1460	89.1 (87.5–90.5)	64,765	93.3 (93.1–93.5)	66,225	93.2 (93.0–93.4)
Any evidence of liver fibrosis	0	0.0 (0.0–0.2)	11	0.0 (0.0–0.0)	11	0.0 (0.0–0.0)
Any history of CC	92	5.6 (4.5–6.8)	2939	4.2 (4.1–4.4)	3031	4.3 (4.1–4.4)
Any history of DLD	87	5.3 (4.3–6.5)	1723	2.5 (2.4–2.6)	1810	2.5 (2.4–2.7)
History of HCC cancer						
No history	1566	95.5 (94.4–96.5)	66,635	96.0 (95.8–96.1)	68,201	96.0 (95.8–96.1)
Any history	73	4.5 (3.5–5.6)	2803	4.0 (3.9–4.2)	2876	4.0 (3.9–4.2)
History of non-HCC cancer						
No history	1575	96.1 (95.0–97.0)	66,395	95.6 (95.5–95.8)	67,970	95.6 (95.5–95.8)
Any history	64	3.9 (3.0–5.0)	3043	4.4 (4.2–4.5)	3107	4.4 (4.2–4.5)
Metabolic syndrome						
No evidence	1496	91.3 (89.8–92.6)	61,670	88.8 (88.6–89.0)	63,166	88.9 (88.6–89.1)
1	124	7.6 (6.3–9.0)	6617	9.5 (9.3–9.8)	6741	9.5 (9.3–9.7)
2	≤18	≤1.1 (−)	≤1060	≤1.5 (−)	1075	1.5 (1.4–1.6)
3	≤4	≤0.2 (−)	≤94	≤0.1 (−)	95	0.1 (0.1–0.2)
Biochemical characteristics at the study entry index date						
HBsAg						
Positive	1525	99.4 (98.9–99.7)	65,626	100 (99.9–100)	67,151	99.9 (99.9–100)
Indeterminate	9	0.6 (0.3–1.1)	26	0.0 (0.0–0.1)	35	0.1 (0.0–0.1)
Missing	105		3786		3891	
HBeAg						
Positive	135	9.1 (7.7–10.6)	12,761	20.4 (20.1–20.8)	12,896	20.2 (19.9–20.5)
Negative	1353	90.9 (89.4–92.3)	49,567	79.4 (79.1–79.7)	50,920	79.6 (79.3–80.0)
Indeterminate	0	0.0 (0.0–0.2)	116	0.2 (0.2–0.2)	116	0.2 (0.1–0.2)
Missing	151		6994		7145	
HBV DNA						
Undetectable	403	24.6 (22.5–26.7)	7976	11.5 (11.3–11.7)	8379	11.8 (11.6–12.0)
Detectable, viral load not available	34	2.1 (1.4–2.9)	999	1.4 (1.4–1.5)	1033	1.5 (1.4–1.5)
Detectable, <2000 IU/mL	861	52.5 (50.1–55.0)	28,888	41.6 (41.2–42.0)	29,749	41.9 (41.5–42.2)
Detectable, 2000–<20,000 IU/mL	91	5.6 (4.5–6.8)	8323	12.0 (11.7–12.2)	8414	11.8 (11.6–12.1)
Detectable, ≥20,000 IU/mL	250	15.3 (13.5–17.1)	23,252	33.5 (33.1–33.8)	23,502	33.1 (32.7–33.4)
ALT ULN						
<1	1082	66.0 (63.7–68.3)	43,332	62.4 (62.0–62.8)	44,414	62.5 (62.1–62.8)
1–<2	297	18.1 (16.3–20.1)	14,954	21.5 (21.2–21.8)	15,251	21.5 (21.2–21.8)
2–<5	121	7.4 (6.2–8.8)	7082	10.2 (10.0–10.4)	7203	10.1 (9.9–10.4)
≥5	139	8.5 (7.2–9.9)	4070	5.9 (5.7–6.0)	4209	5.9 (5.7–6.1)
Treatment at study entry index date						
Untreated, yes	1191	72.7 (70.4–74.8)	45,675	65.8 (65.4–66.1)	46,866	65.9 (65.6–66.3)
IFN monotherapy	≤4	≤0.2 (−)	≤141	≤0.2 (−)	142	0.2 (0.2–0.2)
IFN-alpha	0	0.0 (0.0–0.2)	0	0.0 (0.0–0.0)	0	0.0 (0.0–0.0)
PEG-IFN	≤4	≤0.2 (−)	≤141	≤0.2 (−)	142	0.2 (0.2–0.2)
NA monotherapy	395	24.1 (22.0–26.2)	21,999	31.7 (31.3–32.0)	22,394	31.5 (31.2–31.8)
Tenofovir disoproxil	19	1.2 (0.7–1.8)	1224	1.8 (1.7–1.9)	1243	1.7 (1.7–1.8)
Tenofovir alafenamide	0	0.0 (0.0–0.2)	8	0.0 (0.0–0.0)	8	0.0 (0.0–0.0)
Entecavir	240	14.6 (13.0–16.4)	17,771	25.6 (25.3–25.9)	18,011	25.3 (25.0–25.7)
Lamivudine	115	7.0 (5.8–8.4)	2000	2.9 (2.8–3.0)	2115	3.0 (2.9–3.1)
Adefovir	14	0.9 (0.5–1.4)	329	0.5 (0.4–0.5)	343	0.5 (0.4–0.5)
Telbivudine	7	0.4 (0.2–0.9)	667	1.0 (0.9–1.0)	674	0.9 (0.9–1.0)
History of IFN before NA monotherapy, yes	≤4	≤0.2 (−)	≤194	≤0.3 (−)	195	0.3 (0.2–0.3)
Combination therapies						
IFN and NAs	0	0.0 (0.0–0.2)	17	0.0 (0.0–0.0)	17	0.0 (0.0–0.0)
NA-only combinations	49	3.0 (2.2–3.9)	1609	2.3 (2.2–2.4)	1658	2.3 (2.2–2.4)
Adefovir + lamivudine	25	1.5 (1.0–2.2)	958	1.4 (1.3–1.5)	983	1.4 (1.3–1.5)
Tenofovir disoproxil + entecavir	5	0.3 (0.1–0.7)	130	0.2 (0.2–0.2)	135	0.2 (0.2–0.2)
Tenofovir disoproxil + lamivudine	≤4	≤0.2 (−)	≤120	≤0.2 (−)	121	0.2 (0.1–0.2)
Adefovir + telbivudine	≤4	≤0.2 (−)	≤72	≤0.1 (−)	73	0.1 (0.1–0.1)
Entecavir + lamivudine	≤4	≤0.2 (−)	≤65	≤0.1 (−)	66	0.1 (0.1–0.1)
Adefovir + lamivudine + telbivudine	≤4	≤0.2 (−)				
History of IFN before NA combination therapy, yes	≤4	≤0.2 (−)	≤29	≤0.0 (−)	30	0.0 (0.0–0.1)

Baseline characteristics were reported before excluding patients for the MSM analyses.

IFN, interferon; PEG-IFN, pegylated interferon; ULN, upper limit of normal.

### Patient Characteristics–Clinical and Virological

Evidence/history of liver fibrosis or cirrhosis (including CC and DLD) was present in 6.8% of patients, and history of HCC in 4.0% (Table 1). More patients who went on to experience HBsAg loss had a history of CC and DLD at baseline (10.9%; 95% confidence interval [CI] 9.4%–12.4%) compared with no-loss patients (6.7%; 95% CI 6.5%–6.9%) (Table 1). Also, 79.6% of patients were HBeAg-negative at baseline, and more loss patients (90.9%; 95% CI 89.4%–92.3%) were HBeAg negative at baseline compared with no-loss patients (79.4%; 95% CI 79.1%–79.7%). HBV DNA levels were undetectable in 11.8% patients.

### Patient Characteristics–Biochemistry and Treatment

ALT levels ≥5 times the upper limit of normal were seen in 5.9% of patients; more in HBsAg loss patients (8.5%; 95% CI 7.2%–9.9%) than no-loss patients (5.9%; 95% CI 5.7%–6.0%). Overall, 36.1% of patients were treated at study entry, and fewer loss patients (27.3%; 95% CI 25.2%–29.5%) than no-loss patients (34.2%; 95% CI 33.9%–34.6%) were treated. NA monotherapy was the most common regimen, used in 31.5% of the overall treated population.

Entecavir was the most commonly used NA (25.3%); lamivudine was used by 3.0% (7.0%; 95% CI 5.8%–8.4% in loss and 2.9%; 95% CI 2.8%–3.0% in no-loss patients). Interferon monotherapy was used rarely (0.2% of patients). Additional baseline biochemistry and patient characteristics are shown in Table A3.

### Association Between HBsAg Loss and Clinical Outcomes

The crude event rates per 1000 person-years for all study outcomes were lower during periods of HBsAg loss compared with periods of no-loss (CC, 2.78 vs 8.91 [rate ratio (RR) 0.31; 95% CI 0.17–0.56]; DLD, 0.92 vs 5.79 [RR 0.16; 95% CI 0.06–0.42]; HCC, 3.17 vs 11.06 [RR 0.29; 95% CI 0.17–0.48]; ACM, 13.91 vs 20.83 [RR 0.67; 95% CI 0.53–0.85]) (Table 2).

**Table 2:** Person-Time and Outcome Event Rate by Exposure (HBsAg Loss) Status

Outcome	Exposure status (number of patients who contributed time)	Events, n (%)	Person-y	Crude rate per 1000 person-y (95% CI)
CC	HBsAg loss (n = 1379)	11 (0.8)	3973.3	2.77 (1.38–4.95)
	No HBsAg loss (n = 66,236)	2795 (4.3)	313,676.1	8.91 (8.58–9.25)
DLD	HBsAg loss (n = 1487)	≤4 (≤0.3)	4345.1	0.92 (0.25–2.36)
	No HBsAg loss (n = 69,267)	≤1940 (≤2.9)	334,437.5	5.79 (5.54–6.06)
HCC	HBsAg loss (n = 1489)	14 (0.9)	4417.1	3.17 (1.73–5.32)
	No HBsAg loss (n = 68,201)	3587 (5.4)	324,359.0	11.06 (10.70–11.43)
All-cause mortality	HBsAg loss (n = 1639)	69 (4.2)	4962.0	13.91 (10.82–17.60)
	No HBsAg loss (n = 71,077)	7152 (10.3)	343,417.1	20.83 (20.35–21.31)

**Figure 3:**
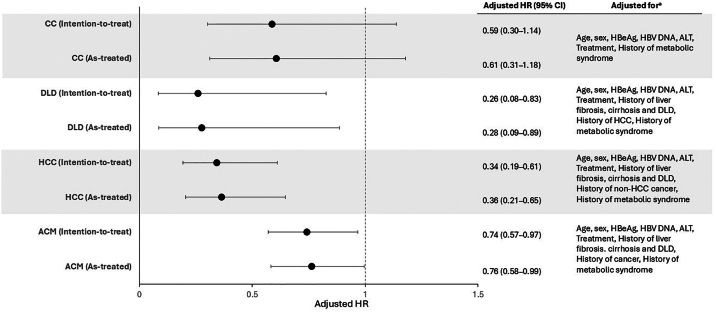
Association of HBsAg loss and outcomes. (a) Variables were modeled as reported in Table A2.

MSM attenuated effect estimates, but HBsAg loss remained associated with a reduction of 74% for DLD (HR 0.26; 95% CI 0.08–0.83), 66% for HCC (HR 0.34; 95% CI 0.19–0.61) and 26% for ACM (HR 0.74; 95% CI 0.57–0.97) (intention-to-treat analysis; Figure 3). Although the point estimate for CC (HR 0.59) suggested HBsAg loss had a beneficial effect, the 95% CI included one (0.30–1.14). Similar results were seen with the as-treated approach (Figure 3), suggesting a minimal impact of seroreversion, which occurred in 27/1639 loss patients. In subgroup analyses, a statistically significant reduction in HCC was observed in patients with no history of CC or DLD (59%, HR 0.41; 95% CI 0.22–0.78), and in ACM for those with no history of DLD (66%, HR 0.34; 95% CI 0.17–0.66).

Each additional month of HBsAg loss was associated with a statistically significant 3.8% decrease in the hazard of HCC (HR 0.962; 95% CI 0.937–0.987) and a 0.9% decrease in the hazard of ACM (HR 0.991; 95% CI 0.985–0.998). Each additional month of HBsAg loss showed a decreased hazard for CC by 1.3% (HR 0.987; 95% CI 0.966–1.008) and a 1.5% decrease in the hazard of DLD (HR 0.985; 95% CI 0.956–1.015), although neither was statistically significant.

### Durability of HBsAg Loss

The cumulative probability of HBsAg loss persistence at 24 (99%) and 60 (97%) months was high (Figure A1). At 24 and 60 months, only 17 (1.0%) and 26 (1.6%) of 1639 loss patients, respectively, experienced seroreversion. Among those who did not experience seroreversion after initial HBsAg loss, 1147 (71.2%) had a further HBsAg test until the end of follow-up, and 1143 (70.9%) had negative tests. In patients with a history and no history of cirrhosis (compensated and decompensated), the cumulative probability of HBsAg loss persistence at 60 months was 93% and 98%, respectively. Persistence was similarly high in males and females, and in patients who were HBeAg-positive or -negative at HBsAg loss. The cumulative probability of loss persistence at 60 months was numerically lower in patients treated with NAs (94% [95% CI 91%–96%]) compared with those untreated (99% [95% CI 98%–100%]) (Figure A2).

### HCRU and HBsAg Loss

**Figure 4:**
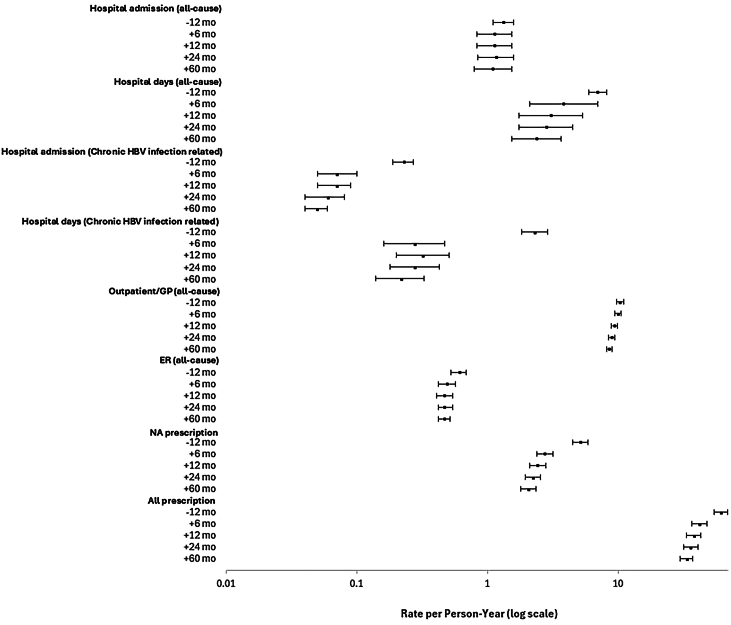
HCRU 1 year prior and up to 5 years after HBsAg loss. ER, emergency room; GP, general practitioner.

HCRU for selected time periods before (1 year) and after (6 months, 1 year, 2 years, and 5 years [or end of follow-up in each]) HBsAg loss are shown in Figure 4, with complete data available in Table A4. The HBsAg loss was associated with a reduction from 0.23 to 0.07 of cHBV infection-related hospital admissions per person-year (PPY) in the 12 months before versus 6 months after HBsAg loss, with this reduction sustained over longer periods. A similar pattern was observed for cHBV infection-related hospital days (2.31 to 0.28 days PPY). HBsAg loss had less of an effect on all-cause hospital admission, but for all-cause hospital days a benefit was observed within 12 months of loss that was sustained over longer time periods. A declining trend in HCRU was observed in the loss population for both outpatient general practitioner and emergency room visits when comparing the 1 year prior to HBsAg loss (10.33 PPY and 0.61 PPY, respectively) with 6 months (9.97 PPY and 0.49 PPY, respectively) or longer after HBsAg loss (5 years, 8.56 PPY and 0.47 PPY, respectively); CIs did not overlap from 24 months and 60 months, respectively (Table A4). Finally, the rate of NA prescribing decreased dramatically after loss; it is not clear how much of this contributed to the observed decline in overall prescribing.

## Discussion

In this large Asian population-based study with an extended follow-up time, HBsAg loss in patients with cHBV infection was associated with a reduced risk of important clinical outcomes, including DLD, HCC, and ACM. These findings are consistent with previous meta-analyses,^8^^,^^12^^,^^16^ in which HBsAg loss was invariably, but not consistently, associated with improved outcomes for HCC, DLD, liver transplantation and/or death, all-cause and liver-related mortality, and cirrhosis. HBsAg loss was also associated with a reduced risk of HCC in a retrospective study of NA-treated patients with chronic hepatitis B in Hong Kong.^13^ These results are consistent with our results for the overall cohort.

We observed a significant risk reduction in HCC among patients with no history of liver complications, and each additional month of HBsAg loss was associated with a reduction in the risk of clinical outcomes as well as ACM. These results suggest that early therapeutic intervention with agents capable of inducing HBsAg loss and viral suppression, may be associated with a reduced risk of HCC in patients with cHBV infection.^13^^,^^17^ A reduced risk of HCC of ∼4% for each additional month of HBsAg loss could, hypothetically, bring clear clinical benefits to patients in relatively short periods (Figure A3), with the hazard approaching zero relative to baseline, assuming a constant risk reduction over time with no plateauing effect. Further data are needed to model and understand appropriately how the risk progressed over time.

We did not find an association between HBsAg loss and reduced risk of CC. Approximately one-third of patients were treated with NAs at study entry; suppressed viral replication in these patients may have decreased the risk of liver damage and thus cirrhosis independently from HBsAg loss.^18^ It is possible that fibrosis status was not adequately captured by International Classification of Diseases codes, leading to underestimation of patients with fibrosis in this study. Severe liver damage in these patients at HBsAg loss may not have been reversible. This is supported by the findings from a prospective matched case-control study where fibrosis regression occurred in a minority of patients experiencing HBsAg loss.^19^ Alternatively, cirrhosis may precede its clinical diagnosis in the natural history of cHBV infection,^20^ and hence the lack of follow-up for this endpoint may have reduced precision around this effect estimate; minimizing bias using the MSM methodology may have compounded this if increased variance widened CIs. A larger sample size may have provided additional statistical power to detect true differences in cirrhosis risk between loss- and no-loss patients, had it existed.

The observed seroreversion in the current study was low. The 97% probability of loss persistence at 60 months is similar to the cumulative rates of HBsAg reversion in spontaneous or NA-induced HBsAg loss over 5 years (1.8%) noted in patients (n = 1972) in Korea, although the seroreversion definition was not reported.^21^ Another CDARS study found no significant difference in the 5-year cumulative probability of confirmed spontaneous and NA-induced HBsAg seroclearance (88.1% vs 92.2%, *P* = .964), defining seroreversion liberally as reappearance of HBsAg after HBsAg seroclearance.^22^

In our study, HBsAg loss occurred on average 74.6 months after study entry. However, this may not accurately reflect time to HBsAg loss from cHBV diagnosis, since most affected patients in Hong Kong are believed to acquire the infection during the perinatal period or childhood.^23^

The significant health-care burden associated with cHBV infection has been documented previously. Umemura et al. reported increased HCRU as disease severity progresses.^24^ Our findings showed a largely consistent trend in decreased HCRU in the HBsAg loss population within the first year after loss compared with the year prior to loss. The HBsAg loss appeared to have a profound and immediate effect on the risk of cHBV infection-related hospital admission, with the 0.23 admissions PPY in the 12 months before loss reduced to 0.07 in the 6 months after, with this sustained over longer periods.

The generalizability of the study results to geographies beyond Hong Kong may be limited by differences between patient populations and health-care systems. CDARS covers most of the Hong Kong population, private care is not captured nor is emigration; nevertheless, patients with chronic diseases may be more likely to use subsidized public health-care services and so the impact of missing private care data is likely limited.

Chronic HBV infection was identified using diagnosis information or laboratory test records. Although the case definition included clinical information relevant to identify patients of interest, the overall validity of case ascertainment remains uncertain. Identifying fibrosis/cirrhosis based solely on codes may result in under-ascertainment due to under-reporting. Research using secondary data sources such as EHRs may also be limited by data completeness and accuracy. Missing data and misclassification can be expected as the underlying routine health-care data were not collected for research purposes.

Our HBsAg loss definition was conservative, which may explain the loss rate (2.3%) observed in our study. Also, study data are from routine health-care records and intervals between follow-up visits were likely to be variable; this may have contributed to underdetection of HBsAg loss. Quantitative HBsAg levels were also not captured in the study and could not be adjusted for at baseline. Thus, misclassification bias may have been introduced as seroreversion events are rare, and it is unlikely that one negative HBsAg test will be followed by a positive test. It is also possible that the association between HBsAg loss and clinical outcomes was influenced by factors such as age and presence of metabolic syndrome; these subgroup analyses were not performed due to sample size considerations, although the association between loss and outcomes was adjusted for age and metabolic syndrome to minimize any impact on results. Further studies with sufficient sample size to enable these subgroups analyses would be of interest, as would analyses according to liver function scores and discontinuation of NA treatment.

Our study also has several strengths. The real-world setting reflects routine clinical practice and comprehensively describes the disease, biochemical characteristics, and associated treatment. The cohort design includes a large number of patients from a territory-wide database covering over 80% of Hong Kong’s population.^13^ The use of MSM and IPW accounted for fixed and time-varying variables allowing adjustment of confounders. Furthermore, the long follow-up provides insights into the long-term outcomes of patients experiencing HBsAg loss.

## Conclusion

In this large population-based study with extended follow-up, HBsAg loss was associated with reduced risk of DLD, HCC, and ACM, and a trend towards decreased HCRU. These findings provide valuable insights into patients with cHBV infection in a real-world setting in Hong Kong, as well as the relationship between HBsAg and improved clinical outcomes in this Asian population.

## Supplementary data

Figure A1

Figure A2

Figure A3

Appendix

Extended PDF

## References

[1] World Health Organization Global hepatitis report 2024: action for access in low- and middle-income countries. https://www.who.int/publications/i/item/9789240091672.

[2] Mak L.Y., Liu K., Chirapongsathorn S. (2024). Liver diseases and hepatocellular carcinoma in the Asia-Pacific region: burden, trends, challenges and future directions. Nat Rev Gastroenterol Hepatol.

[3] The Government of the Hong Kong Special Administrative Region Department of Health PHS 2020-22 – thematic report on viral hepatitis. https://www.hepatitis.gov.hk/english/health_professionals/files/Thematic_Report_on_Viral_Hepatitis_Executive_summary.pdf.

[4] Wong N.S., Chan D.P.C., Poon C.M. (2023). Hepatitis B burden and population immunity in a high endemicity city - a geographically random household epidemiology study for evaluating achievability of elimination. Epidemiol Infect.

[5] Liaw Y.F. (2019). Clinical utility of HBV surface antigen quantification in HBV e antigen-negative chronic HBV infection. Nat Rev Gastroenterol Hepatol.

[6] Song J.E., Kim D.Y. (2016). Diagnosis of hepatitis B. Ann Transl Med.

[7] Ye B., Liu X., Li X. (2015). T-cell exhaustion in chronic hepatitis B infection: current knowledge and clinical significance. Cell Death Dis.

[8] Anderson R.T., Choi H.S.J., Lenz O. (2021). Association between seroclearance of hepatitis B surface antigen and long-term clinical outcomes of patients with chronic hepatitis B virus infection: systematic review and meta-analysis. Clin Gastroenterol Hepatol.

[9] Zhou K., Contag C., Whitaker E. (2019). Spontaneous loss of surface antigen among adults living with chronic hepatitis B virus infection: a systematic review and pooled meta-analyses. Lancet Gastroenterol Hepatol.

[10] Ghany M.G., Buti M., Lampertico P. (2023). Guidance on treatment endpoints and study design for clinical trials aiming to achieve cure in chronic hepatitis B and D: report from the 2022 AASLD-EASL HBV-HDV treatment endpoints conference. Hepatology.

[11] Zheng J., Wang Z., Huang L. (2025). Achieving chronic hepatitis B functional cure: factors and potential mechanisms. Virus Res.

[12] Morais E., Mason L., Dever J. (2023). Clinical consequences of hepatitis B surface antigen loss in chronic hepatitis B infection: a systematic literature review and meta-analysis. Gastro Hep Adv.

[13] Yip T.C., Wong G.L., Chan H.L. (2019). HBsAg seroclearance further reduces hepatocellular carcinoma risk after complete viral suppression with nucleos(t)ide analogues. J Hepatol.

[14] Wu D.R., Nam R., Leung K.S.K. (2023). Population-based clinical studies using routinely collected data in Hong Kong, China: a systematic review of trends and established local practices. Cardiovasc Innov Appl.

[15] D’Agostino R.B., Lee M.L., Belanger A.J. (1990). Relation of pooled logistic regression to time dependent Cox regression analysis: the Framingham Heart Study. Stat Med.

[16] Vittal A., Sharma D., Hu A. (2022). Systematic review with meta-analysis: the impact of functional cure on clinical outcomes in patients with chronic hepatitis B. Aliment Pharmacol Ther.

[17] Lim Y.S., Kim W.R., Dieterich D. (2023). Evidence for benefits of early treatment initiation for chronic hepatitis B. Viruses.

[18] Broquetas T., Carrion J.A. (2022). Current perspectives on nucleos(t)ide analogue therapy for the long-term treatment of hepatitis B virus. Hepat Med.

[19] Mak L.Y., Hui R.W., Chung M.S.H. (2024). Regression of liver fibrosis after HBsAg loss: a prospective matched case-control evaluation using transient elastography and serum enhanced liver fibrosis test. J Gastroenterol Hepatol.

[20] Pu C., Zhen W., Wenchong Z. (2025). Identifying liver cirrhosis in patients with chronic hepatitis B: an interpretable machine learning algorithm based on LSM. Ann Med.

[21] Choi J., Yoo S., Lim Y.S. (2021). Comparison of long-term clinical outcomes between spontaneous and therapy-induced HBsAg seroclearance. Hepatology.

[22] Yip T.C., Wong G.L., Wong V.W. (2018). Durability of hepatitis B surface antigen seroclearance in untreated and nucleos(t)ide analogue-treated patients. J Hepatol.

[23] The Government of the Hong Kong Special Administrative Region Department of Health Management of adult patients with chronic hepatitis B in primary care. https://www.hepatitis.gov.hk/english/health_professionals/files/Management_of_Adult_Patients_with_CHB_in_Primary_Care_full_guidance.pdf.

[24] Umemura T., Wattanakamolkul K., Nakayama Y. (2023). Real-world epidemiology, clinical and economic burden of chronic hepatitis B in Japan: a retrospective study using JMDC claims database. Infect Dis Ther.

