# Supplementary figures and images for: Hepatitis B Surface Antigen Loss and Improved Clinical Outcomes in Asians with Chronic Hepatitis B Virus Infection
Source: Gastro Hep Adv. 2025 Nov 6;5(2):100844. doi: 10.1016/j.gastha.2025.100844 (PMC12757638; doi:10.1016/j.gastha.2025.100844)

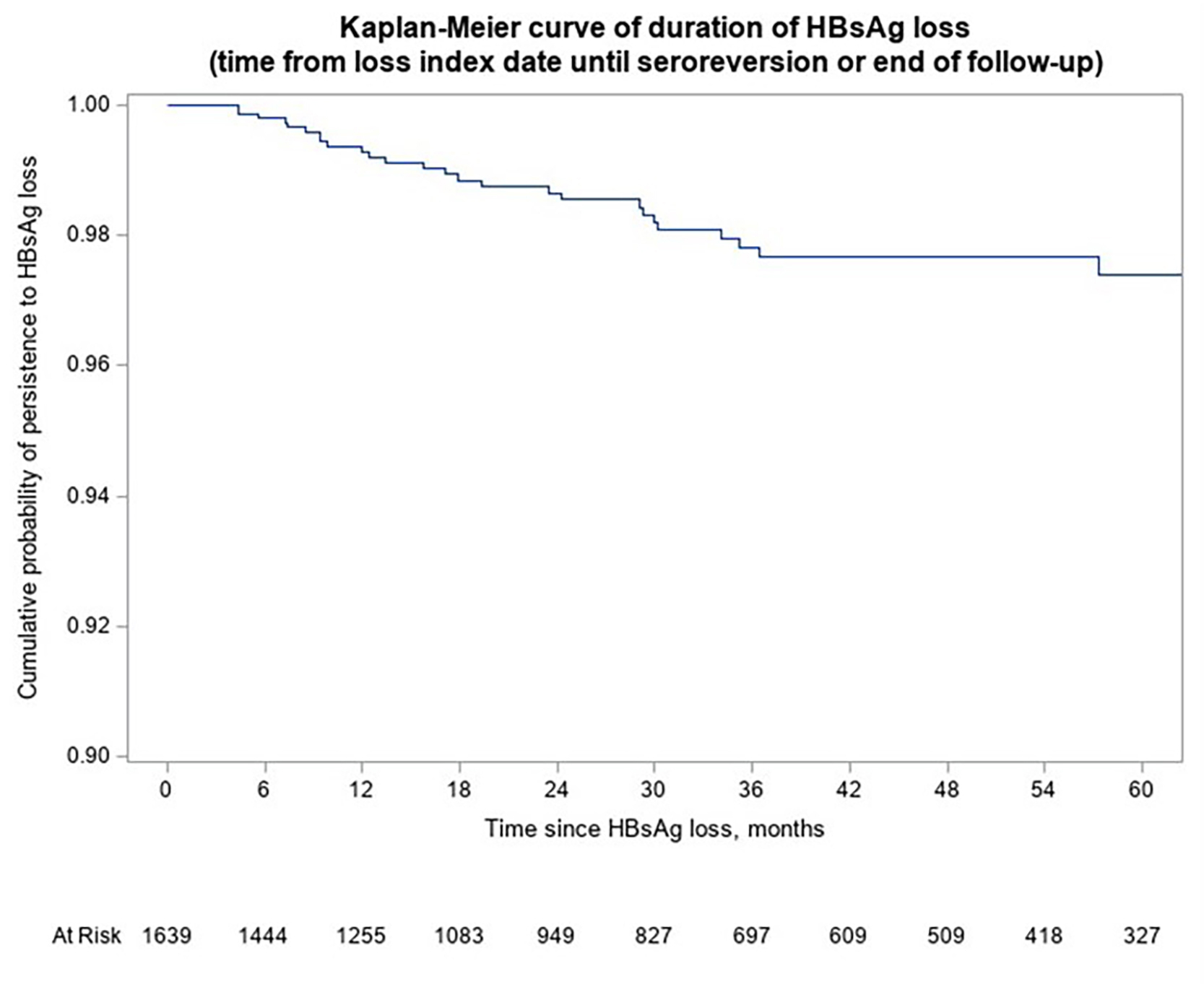

Supplement: Figure A1 [file figs1.jpg]

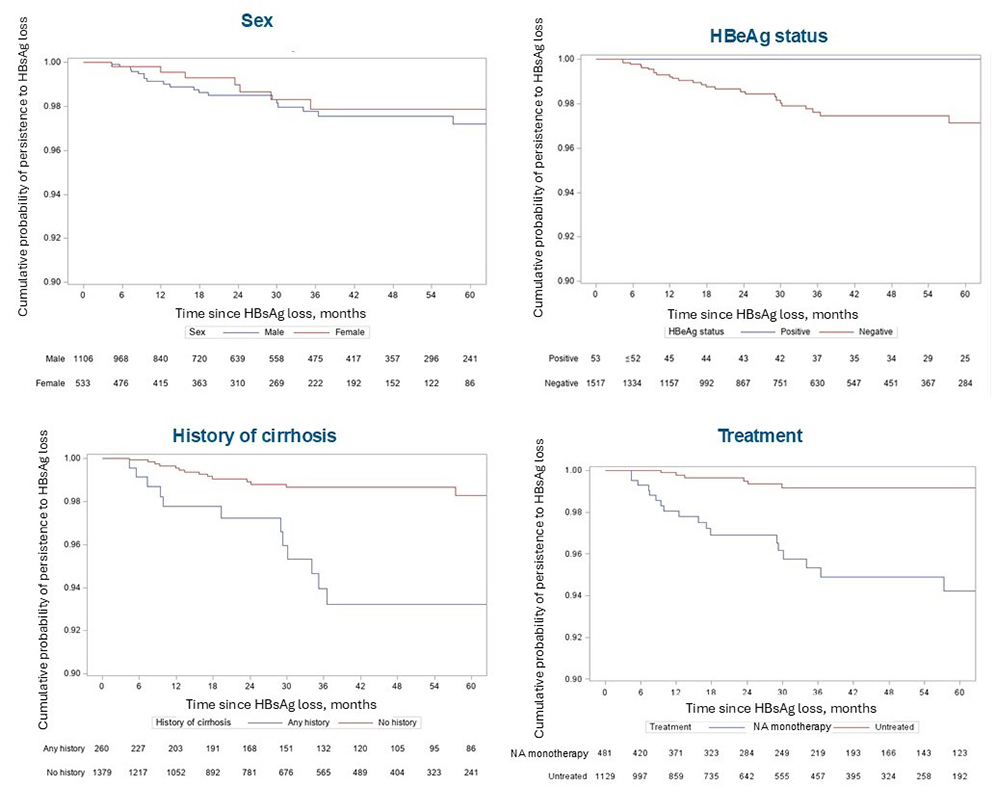

Supplement: Figure A2 [file figs2.jpg]

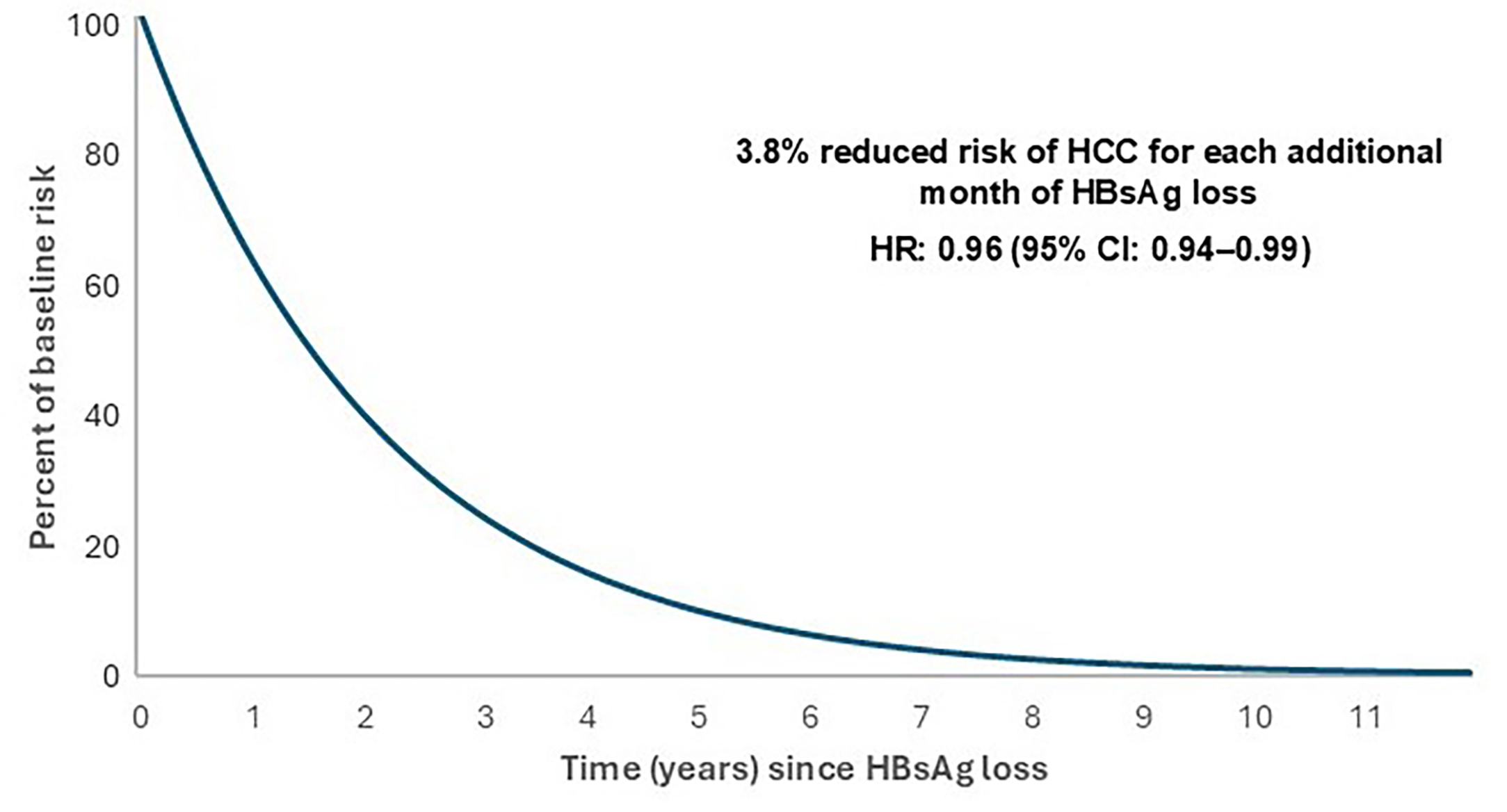

Supplement: Figure A3 [file figs3.jpg]
