# Supplementary material for: Hepatitis B Surface Antigen Loss and Improved Clinical Outcomes in Asians with Chronic Hepatitis B Virus Infection
Source: Gastro Hep Adv. 2025 Nov 6;5(2):100844. doi: 10.1016/j.gastha.2025.100844 (PMC12757638; doi:10.1016/j.gastha.2025.100844)

Supplementary Material

Hepatitis B Surface Antigen Loss and Improved Clinical Outcomes in Asians With Chronic Hepatitis B Virus Infection

Short title: HBsAg loss and improved clinical outcomes

Wallis Lau,[1,2] Myriam Drysdale,[3] Eleonora Morais,[3] Luis Antunes,[4] Loey Mak,[5] Christopher Lee,[6] Catarina Camarinha,[4] Xiaohui Sun,[6] Adrienne Y.L. Chan,[1,7] May Lam,[1] Vera Gielen,[3] Dickens Theodore,[8] Ian Wong,[1] Iain A. Gillespie[9]

[1]Department of Pharmacology and Pharmacy, The University of Hong Kong, Hong Kong

[2]UCL School of Pharmacy, London, UK

[3]GSK, London, UK

[4]IQVIA, Lisbon, Portugal

[5]Department of Medicine, The University of Hong Kong, Hong Kong

[6]IQVIA, London, UK

[7]Aston Pharmacy School, Aston University, Birmingham, UK

[8]GSK, Durham, NC, USA

[9]GSK, Stevenage, UK

Corresponding author: Myriam Drysdale, GSK, 79 New Oxford St, London WC1A 1DG; Email: myriam.g.drysdale@gsk.com; Telephone number:+447443594081

Eligibility criteria

Inclusion:

- Patient meets the case definition of chronic HBV infection with first evidence within the identification period
- Patient is alive and ≥18 years of age at study entry index date
- Patient has ≥1 valid record for both HBV DNA and ALT during the overall study period (1 January 2000 to 31 December 2019) and before the HBsAg loss index date

Exclusion:

- Missing data on age and/or sex
- ≥1 negative laboratory result for HBsAg prior to or on the study entry index date, including individuals who serocleared and subsequently seroreverted at baseline
- Co-infection with hepatitis C virus or hepatitis D virus prior to or on the study entry index date
- Co-infection with human immunodeficiency virus at any point in the study period
- Receipt of immunosuppressives prior to or on the study entry index date

The International Classification of Diseases, Ninth Revision codes used to identify the covariates, outcomes, and inclusion/exclusion criteria are listed in **Supplementary Table 1**. Statistical analyses were conducted using SAS version 9.4 (SAS Institute).

Subgroup analyses

Results from NA-treated patients

A total of 15,300 patients were in the NA-treated sub-group, of whom 267 (1.7%) experienced HBsAg loss and 15,033 (98.3%) did not. At study entry index date, the majority of NA-treated patients were receiving NA monotherapy, n=13,899 (90.8%); 1401 (9.2%) were receiving NA combination therapy. Entecavir was the most common NA treatment (70.0%), followed by lamivudine (10.8%), tenofovir disoproxil (5.6%), and telbivudine (2.4%).

Evidence of liver fibrosis or cirrhosis was present in 24.3% (95%CI: 25.0–23.6%) of NA-treated patients, and a history of HCC in 8.9%. Similar to the overall group, 75.1% of NA-treated patients were negative for HBeAg and 37.1% had undetectable HBV DNA.

HBsAg loss was associated with a significant 51% decrease in the hazard of ACM (HR 0.49, 95%CI: 0.26–0.93). A decreased hazard of CC by 68% and HCC by 38% was also present, although not statistically significant. Association of HBsAg loss and DLD was not assessed due to the lack of patients experiencing DLD.

Supplementary Table A1. ICD-9-CM codes used in the study

ICD9-CM codes	Code description
**Chronic HBV**	
070.22	Chronic viral hepatitis B with hepatic coma without hepatitis delta
070.23	Chronic viral hepatitis B with hepatic coma with hepatitis delta
070.32	Chronic viral hepatitis B without mention of hepatic coma without mention of hepatitis delta
070.33	Chronic viral hepatitis B without mention of hepatic coma with hepatitis delta
**HCV/HDV/HIV**	
070.41	Acute hepatitis C with hepatic coma
070.44	Chronic hepatitis C with hepatic coma
070.51	Acute hepatitis C without mention of hepatic coma
070.54	Chronic hepatitis C without mention of hepatic coma
070.70	Unspecified viral hepatitis C without hepatic coma
070.71	Unspecified viral hepatitis C with hepatic coma
V02.62	Hepatitis C carrier
070.21	Viral hepatitis B with hepatic coma, acute or unspecified, with hepatitis delta
070.23	Chronic viral hepatitis B with hepatic coma with hepatitis delta
070.31	Viral hepatitis B without mention of hepatic coma, acute or unspecified, with hepatitis delta
070.33	Chronic viral hepatitis B without mention of hepatic coma with hepatitis delta
070.42	Hepatitis delta without mention of active hepatitis B disease with hepatic coma
070.52	Hepatitis delta without mention of active hepatitis B disease or hepatic coma
**Fibrosis**	
571.9	Unspecified chronic liver disease without mention of alcohol
**Compensated cirrhosis**	
571.2	Alcoholic cirrhosis of liver
571.5	Cirrhosis of liver without mention of alcohol
**Decompensated liver disease**	
572.2	Hepatic encephalopathy
456.0	Esophageal varices with bleeding
456.20	Esophageal varices in diseases classified elsewhere, with bleeding
789.5	Ascites
789.59	Other ascites
070.2	Viral hepatitis B with hepatic coma
070.20	Viral hepatitis B with hepatic coma, acute or unspecified, without mention of hepatitis delta
070.21	Viral hepatitis B with hepatic homa, acute or unspecified, with hepatitis delta
070.22	Chronic viral hepatitis B with hepatic coma without hepatitis delta
070.23	Chronic viral hepatitis B with hepatic coma with hepatitis delta
070.4	Other specified viral hepatitis with hepatic coma
070.41	Acute hepatitis C with hepatic coma
070.42	Hepatitis delta without mention of active hepatitis B disease with hepatic coma
070.43	Hepatitis E with hepatic coma
070.44	Chronic hepatitis C with hepatic coma
070.49	Other specified viral hepatitis with hepatic coma
070.6	Unspecified viral hepatitis with hepatic coma

070.71	Unspecified viral hepatitis C with hepatic coma
070.0	Acute hepatitis A with hepatic coma
**HCC**	
155.0	Malignant neoplasm of liver, primary
155.2	Malignant neoplasm of liver, not specified as primary or secondary

IFN medication

ATC code	BNF code	Name	DDD	Unit	Route of administration
L03AB04	0802040J0	interferon alfa-2a	2	MU	parenteral
L03AB05	0802040M0	interferon alfa-2b	2	MU	parenteral
L03AB10	0802040AP	peginterferon alfa-2b	7.5	mcg	parenteral
L03AB11	0802040A0	peginterferon alfa-2a	26	mcg	parenteral

NA medication

ATC code	BNF code	Name	DDD	Unit	Route of administration
J05AF05	0503010Q0	lamivudine	0.3	g	oral
J05AF11	0503031C0	telbivudine	0.6	g	oral
J05AF11	0503030D0	telbivudine			
J05AF08	0503031A0	adefovir dipivoxil	10	mg	oral
	0503030B0	adefovir dipivoxil			
J05AF10	0503030C0	entecavir	0.5	mg	oral
	0503031B0	entecavir			
J05AF07	0503010H0	tenofovir disoproxil	0.245	g	oral
J05AF13	0503031D0	tenofovir alafenamide	25	mg	oral

ATC, Anatomical Therapeutic Chemical; BNF, British National Formulary; DDD, Defined Daily Dose; HBV, hepatitis B virus; HCC, hepatocellular carcinoma; HCV, hepatitis C virus; HDV, hepatitis D virus; HIV, human immunodeficiency virus; ICD-9-CM, International Classification of Diseases, 9th Revision, Clinical Modification; IFN, interferon; mcg, microgram; MU, million units.

Supplementary Table A2. Variables adjusted for in the study

Variables	Definitions
**Time-fixed variables**	
Sex	Male<br>Female<br>Reference category in regression models: Female
HBeAg evidence of positivity at baseline	Negative: No, indeterminate and missing<br>Positive: Yes<br>Reference category in regression models: Negative
HBV DNA status at baseline	Undetectable<br><2000 / viral load not available<br>≥2000<br>Reference category in regression models: Undetectable
ALT ULN	<1<br>1–<2<br>≥2[a]<br>Reference category in regression models: <1 × ALT × ULN<br><br>A fixed value for ULN of 40 IU/L was used to calculate the ALT × ULN value (ALT result / 40)
History of liver fibrosis and cirrhosis at baseline	No evidence/history of liver fibrosis or cirrhosis<br>Any evidence of liver fibrosis/Any history of compensated cirrhosis/<br>Any history of decompensated liver disease<br>Reference category in regression models: No history/evidence of each condition
History of cancer at baseline	Reference category in regression models: No history
History of metabolic syndrome at baseline (type 2 diabetes mellitus, hyperlipidemia, overweight/obesity)	No evidence<br>≥1<br>Reference category in regression models: No evidence
Treatment at baseline	Untreated<br>Treated<br>Reference category in regression models: Untreated
**Time-varying variables**	
Age	Continuous time-varying variable

**Laboratory values**	
HBeAg evidence of positivity over time	Negative: No, indeterminate and missing<br>Positive: Yes<br>Reference category in regression models: Negative
HBV DNA status over time	Undetectable<br><2000 / viral load not available<br>≥2000<br>Reference category in regression models: Undetectable
ALT ULN over time	<1<br>1–<2<br>≥2[a]<br>Reference category in regression models: <1 × ALT × ULN<br><br>A fixed value for ULN of 40 IU/L was used to calculate the ALT × ULN value (ALT result / 40)
**Clinical history variables**	
Liver fibrosis and cirrhosis over time	No evidence/history of liver fibrosis or cirrhosis<br>Any evidence of liver fibrosis/Any history of compensated cirrhosis/<br>Any history of decompensated liver disease<br>Reference category in regression models: No history/evidence of each condition
Cancer over time	No history<br>Any history<br>Reference category in regression models: No history
Metabolic syndrome over time (type 2 diabetes mellitus, hyperlipidemia, overweight/obesity)	No evidence<br>≥1<br>Reference category in regression models: No evidence
Treatment over time<br>NA monotherapy<br>IFN monotherapy<br>IFN and NA combination therapy<br>NA combination therapy	Untreated<br>Treated<br>Reference category in regression models: Untreated<br><br>• Untreated: any patient with no recorded prescriptions of any IFN and no prescriptions of any NA medication on the study entry index date<br>• NA monotherapy: any patient with ≥1 recorded prescription of only 1 type of NA and no concurrent prescriptions of IFN medication and/or other NA(s) on the study entry index date<br>• IFN monotherapy: any patient with ≥1 recorded prescription of any IFN and no

	concurrent prescriptions of NA medication on the study entry index date<br>• IFN and NA combination therapy: any patient with ≥1 recorded prescription of any IFN and ≥ 1 recorded concurrent prescription of any NA medication on the study entry index date<br>NA combination therapy: any patient with ≥2 concurrent recorded prescriptions of NAs and no concurrent prescriptions of any IFN medication on the study entry index date

[a]2–<5 and ≥5 combined into one category.

ALT, alanine aminotransferase; HBeAg, hepatitis B e antigen; HBsAg, hepatitis B surface antigen; HBV, hepatitis B virus; HCC, hepatocellular carcinoma; IFN, interferon; NA, nucleos(t)ide analogue; PEG-IFN, pegylated interferon; ULN, upper limit of normal.

Supplementary Table A3. Full baseline characteristics

	HBsAg loss N=1639		No HBsAg loss N=69,438		Overall population N=71,077	
	**N**	**% (95%CI)**	**N**	**% (95%CI)**	**N**	**% (95%CI)**
**Demographics at study entry index date**						
Age (continuous, in years)						
N (%)	1639 (100)		69,438 (100)		71,077 (100)	
Mean (SD)	52.51 (11.07)		52.74 (13.73)		52.73 (13.67)	
Median (Q1–Q3)	53 (46–60)		53 (43–62)		53 (43–62)	
Min–Max	20–83		18–103		18–103	
Age (categorical, in years)						
18–<30	44	2.7 (2.0–3.6)	3140	4.5 (4.4–4.7)	3184	4.5 (4.3–4.6)
30–<40	175	10.7 (9.2–12.3)	9923	14.3 (14.0–14.6)	10,098	14.2 (14.0–14.5)
40–<50	365	22.3 (20.3–24.4)	14,195	20.4 (20.1–20.7)	14,560	20.5 (20.2–20.8)
50–<60	633	38.6 (36.3–41.0)	20,601	29.7 (29.3–30.0)	21,234	29.9 (29.5–30.2)
60–<70	333	20.3 (18.4–22.3)	14,099	20.3 (20.0–20.6)	14,432	20.3 (20.0–20.6)
70+	89	5.4 (4.4–6.6)	7480	10.8 (10.5–11.0)	7569	10.6 (10.4–10.9)
Sex						
Female	533	32.5 (30.3–34.8)	29,306	42.2 (41.8–42.6)	29,839	42.0 (41.6–42.3)
Male	1106	67.5 (65.2–69.7)	40,132	57.8 (57.4–58.2)	41,238	58.0 (57.7–58.4)
**Time since chronic HBV infection index date**						
Time since index date (in months) at study entry index date						
N	1639 (100)		69438 (100)		71077 (100)	
Mean (SD)	23.92 (27.75)		21.88 (32.48)		21.93 (32.38)	
Median (Q1–Q3)	15.03 (0–38.29)		2.99 (0–34.21)		3.22 (0–34.31)	
Min–Max	0–143.85		0–176.55		0–176.55	

Time since index date (in months) at loss index date (HBsAg loss group only)						
N	1639 (100)		NA	NA	NA	NA
Mean (SD)	74.63 (37.52)		NA	NA	NA	NA
Median (Q1–Q3)	72.37 (45.36–101.74)		NA	NA	NA	NA
Min–Max	6.22–177.93		NA	NA	NA	NA
Time since index date (categorical, in months) at loss index date (HBsAg loss group only)						
<6	0	0.0 (0.0–0.2)	NA	NA	NA	NA
6–<12	44	2.7 (2.0–3.6)	NA	NA	NA	NA
12–<18	52	3.2 (2.4–4.1)	NA	NA	NA	NA
18–<24	55	3.4 (2.5–4.3)	NA	NA	NA	NA
24–<36	138	8.4 (7.1–9.9)	NA	NA	NA	NA
36–<48	168	10.3 (8.8–11.8)	NA	NA	NA	NA
48–<60	162	9.9 (8.5–11.4)	NA	NA	NA	NA
60+	1020	62.2 (59.8–64.6)	NA	NA	NA	NA
**Clinical history at the study entry index date**						
Liver fibrosis and cirrhosis						
No evidence/history of liver fibrosis or cirrhosis	1460	89.1 (87.5–90.5)	64,765	93.3 (93.1–3.5)	66,225	93.2 (93.0–93.4)
Any evidence of liver fibrosis	0	0.0 (0.0–0.2)	11	0.0 (0.0–0.0)	11	0.0 (0.0–0.0)
Any history of compensated cirrhosis	92	5.6 (4.5–6.8)	2939	4.2 (4.1–4.4)	3031	4.3 (4.1–4.4)
Any history of decompensated liver disease	87	5.3 (4.3–6.5)	1723	2.5 (2.4–2.6)	1810	2.5 (2.4–2.7)
History of HCC cancer						
No history	1566	95.5 (94.4–96.5)	66,635	96.0 (95.8–96.1)	68,201	96.0 (95.8–96.1)
Any history	73	4.5 (3.5–5.6)	2803	4.0 (3.9–4.2)	2876	4.0 (3.9–4.2)
History of non-HCC cancer						
No history	1575	96.1 (95.0–97.0)	66,395	95.6 (95.5–95.8)	67,970	95.6 (95.5–95.8)
Any history	64	3.9 (3.0–5.0)	3043	4.4 (4.2–4.5)	3107	4.4 (4.2–4.5)

History of liver transplant						
No history	1639	100 (99.8–100)	≤69,437	≤100 (-)	≤71,076	≤100 (-)
Any history	0	0.0 (0.0–0.2)	≤4	≤0.0 (-)	≤4	≤0.0 (-)
History of overweight/obesity						
No evidence	1625	99.1 (98.6–99.5)	68,800	99.1 (99.0–99.2)	70,425	99.1 (99.0–99.2)
Any evidence overweight	0	0.0 (0.0–0.2)	0	0.0 (0.0–0.0)	0	0.0 (0.0–0.0)
Any evidence obesity	14	0.9 (0.5–1.4)	638	0.9 (0.8–1.0)	652	0.9 (0.8–1.0)
History of type 2 diabetes mellitus						
No evidence	1520	92.7 (91.4–93.9)	63,306	91.2 (91.0–91.4)	64,826	91.2 (91.0–91.4)
Any evidence	119	7.3 (6.1–8.6)	6132	8.8 (8.6–9.0)	6251	8.8 (8.6–9.0)
History of hyperlipidemia						
No evidence	1609	98.2 (97.4–98.8)	67,195	96.8 (96.6–96.9)	68,804	96.8 (96.7–96.9)
Any evidence	30	1.8 (1.2–2.6)	2243	3.2 (3.1–3.4)	2273	3.2 (3.1–3.3)
Metabolic syndrome						
No evidence	1496	91.3 (89.8–92.6)	61,670	88.8 (88.6–89.0)	63,166	88.9 (88.6–89.1)
1	124	7.6 (6.3–9.0)	6617	9.5 (9.3–9.8)	6741	9.5 (9.3–9.7)
2	≤18	≤1.1 (-)	≤1060	≤1.5 (-)	1075	1.5 (1.4–1.6)
3	≤4	≤0.2 (-)	≤94	≤0.1 (-)	95	0.1 (0.1–0.2)
History of CKD						
No evidence	1622	99.0 (98.3–99.4)	68,462	98.6 (98.5–98.7)	70,084	98.6 (98.5–98.7)
Any evidence – chronic kidney disease on dialysis	6	0.4 (0.1-0.8)	307	0.4 (0.4–0.5)	313	0.4 (0.4–0.5)
Any evidence – chronic kidney disease not on dialysis	11	0.7 (0.3–1.2)	669	1.0 (0.9–1.0)	680	1.0 (0.9–1.0)
History of autoimmune hepatitis						
No history	1639	100 (99.8–100)	69,438	100 (100–100)	71,077	100 (100–100)
Any history	0	0.0 (0.0–0.2)	0	0.0 (0.0–0.0)	0	0.0 (0.0–0.0)
History of hereditary hemochromatosis						
No history	1639	100 (99.8–100)	69,438	100 (100–100)	71,077	100 (100–100)
Any history	0	0.0 (0.0–0.2)	0	0.0 (0.0–0.0)	0	0.0 (0.0–0.0)

History of alpha-I antitrypsin deficiency						
No history	1639	100 (99.8–100)	69,438	100 (100–100)	71,077	100 (100–100)
Any history	0	0.0 (0.0–0.2)	0	0.0 (0.0–0.0)	0	0.0 (0.0–0.0)
History of Wilson's disease						
No history	≤1638	≤99.9 (-)	≤69,437	≤100 (-)	≤71,076	≤100 (-)
Any history	≤4	≤0.2 (-)	≤4	≤0.0 (-)	≤4	≤0.0 (-)
History of primary biliary cholangitis						
No history	1626	99.2 (98.6–99.6)	69,017	99.4 (99.3–99.5)	70,643	99.4 (99.3–99.4)
Any history	13	0.8 (0.4–1.4)	421	0.6 (0.5–0.7)	434	0.6 (0.6–0.7)
History of NALD						
No evidence	1619	98.8 (98.1–99.3)	68,844	99.1 (99.1–99.2)	70,463	99.1 (99.1–99.2)
Any evidence	20	1.2 (0.7–1.9)	594	0.9 (0.8–0.9)	614	0.9 (0.8–0.9)
History of alcoholic liver disease						
No evidence	≤1638	≤99.9 (-)	≤69,325	≤99.8 (-)	70,960	99.8 (99.8–99.9)
Any evidence	≤4	≤0.2 (-)	≤116	≤0.2 (-)	117	0.2 (0.1–0.2)
History of alcoholism						
No evidence	≤1638	≤99.9 (-)	≤69,365	≤99.9 (-)	71,000	99.9 (99.9–99.9)
Any evidence	≤4	≤0.2 (-)	≤76	≤0.1 (-)	77	0.1 (0.1–0.1)
**Biochemical characteristics at the study entry index date**						
HBsAg						
Positive	1525	99.4 (98.9–99.7)	65,626	100 (99.9–100)	67,151	99.9 (99.9–100)
Indeterminate	9	0.6 (0.3–1.1)	26	0.0 (0.0–0.1)	35	0.1 (0.0–0.1)
Missing	105		3786		3891	
HBeAg						
Positive	135	9.1 (7.7–10.6)	12,761	20.4 (20.1–20.8)	12,896	20.2 (19.9–20.5)
Negative	1353	90.9 (89.4–92.3)	49,567	79.4 (79.1–79.7)	50,920	79.6 (79.3–80.0)
Indeterminate	0	0.0 (0.0–0.2)	116	0.2 (0.2–0.2)	116	0.2 (0.1–0.2)
Missing	151		6994		7145	
HBV DNA						

Undetectable	403	24.6 (22.5–26.7)	7976	11.5 (11.3–11.7)	8379	11.8 (11.6–12.0)
Detectable, viral load not available	34	2.1 (1.4–2.9)	999	1.4 (1.4–1.5)	1033	1.5 (1.4–1.5)
Detectable, <2000 IU/mL	861	52.5 (50.1–55.0)	28,888	41.6 (41.2–42.0)	29,749	41.9 (41.5–42.2)
Detectable, 2000–<20,000 IU/mL	91	5.6 (4.5–6.8)	8323	12.0 (11.7–12.2)	8414	11.8 (11.6–12.1)
Detectable, ≥20,000 IU/mL	250	15.3 (13.5–17.1)	23,252	33.5 (33.1–33.8)	23,502	33.1 (32.7–33.4)
ALT ULN						
<1	1082	66.0 (63.7–68.3)	43,332	62.4 (62.0–62.8)	44,414	62.5 (62.1–62.8)
1–<2	297	18.1 (16.3–20.1)	14,954	21.5 (21.2–21.8)	15,251	21.5 (21.2–21.8)
2–<5	121	7.4 (6.2–8.8)	7082	10.2 (10.0–10.4)	7203	10.1 (9.9–10.4)
≥5	139	8.5 (7.2–9.9)	4070	5.9 (5.7–6.0)	4209	5.9 (5.7–6.1)
**Treatment at study entry index date**						
Untreated, yes	1191	72.7 (70.4–74.8)	45,675	65.8 (65.4–66.1)	46,866	65.9 (65.6–66.3)
IFN monotherapy	≤4	≤0.2 (-)	≤141	≤0.2 (-)	142	0.2 (0.2–0.2)
IFN-alpha	0	0.0 (0.0–0.2)	0	0.0 (0.0-0.0)	0	0.0 (0.0–0.0)
PEG-IFN	≤4	≤0.2 (-)	≤141	≤0.2 (-)	142	0.2 (0.2–0.2)
NA monotherapy	395	24.1 (22.0–26.2)	21,999	31.7 (31.3–32.0)	22,394	31.5 (31.2–31.8)
Tenofovir disoproxil	19	1.2 (0.7–1.8)	1224	1.8 (1.7–1.9)	1243	1.7 (1.7–1.8)
Tenofovir alafenamide	0	0.0 (0.0–0.2)	8	0.0 (0.0–0.0)	8	0.0 (0.0–0.0)
Entecavir	240	14.6 (13.0–16.4)	17,771	25.6 (25.3–25.9)	18,011	25.3 (25.0–25.7)
Lamivudine	115	7.0 (5.8–8.4)	2000	2.9 (2.8–3.0)	2115	3.0 (2.9–3.1)
Adefovir	14	0.9 (0.5–1.4)	329	0.5 (0.4–0.5)	343	0.5 (0.4–0.5)
Telbivudine	7	0.4 (0.2–0.9)	667	1.0 (0.9–1.0)	674	0.9 (0.9–1.0)
History of IFN before NA monotherapy, yes	≤4	≤0.2 (-)	≤194	≤0.3 (-)	195	0.3 (0.2–0.3)
Combination therapies						
IFN and NAs	0	0.0 (0.0–0.2)	17	0.0 (0.0–0.0)	17	0.0 (0.0–0.0)
NA only combinations	49	3.0 (2.2–3.9)	1609	2.3 (2.2–2.4)	1658	2.3 (2.2–2.4)
Adefovir + lamivudine	25	1.5 (1.0–2.2)	958	1.4 (1.3–1.5)	983	1.4 (1.3–1.5)
Tenofovir disoproxil + entecavir	5	0.3 (0.1–0.7)	130	0.2 (0.2–0.2)	135	0.2 (0.2–0.2)
Tenofovir disoproxil + lamivudine	≤4	≤0.2 (-)	≤120	≤0.2 (-)	121	0.2 (0.1–0.2)

Adefovir + telbivudine	≤4	≤0.2 (-)	≤72	≤0.1 (-)	73	0.1 (0.1–0.1)
Entecavir + lamivudine	≤4	≤0.2 (-)	≤65	≤0.1 (-)	66	0.1 (0.1–0.1)
Adefovir + lamivudine + telbivudine	≤4	≤0.2 (-)				
History of IFN before NA combination therapy, yes	≤4	≤0.2 (-)	≤29	≤0.0 (-)	30	0.0 (0.0–0.1)

Baseline characteristics were reported before excluding patients for the MSM analyses.

ALT, alanine aminotransferase; HBeAg, hepatitis B e antigen; HBsAg, hepatitis B surface antigen; HBV, hepatitis B virus; HCC, hepatocellular carcinoma; IFN, interferon; MSM, marginal structural modelling; NA, nucleos(t)ide analogue; PEG-IFN, pegylated interferon; Q, quartile; SD, standard deviation; ULN, upper limit of normal.

Supplementary Table A4. HCRU 1 year prior and up to 5 years after HBsAg loss

		12-months prior HBsAg loss		6-months post-loss or end of follow-up		12-months post-loss or end of follow-up		24-months post-loss or end of follow-up		60-months post-loss or end of follow-up	
		Overall	Sum	Overall	Sum	Overall	Sum	Overall	Sum	Overall	Sum
		N	%	N	%	N	%	N	%	N	%
Hospital admissions (all-cause)	Patients with events	507	30.9%	250	15.3%	361	22.0%	477	29.1%	613	37.4%
	Count of events	2155		863		1639		2936		4828	
	Rate PPY	1.33		1.13		1.13		1.16		1.1	
	95%CI of rate	1.11–1.58		0.83–1.53		0.83–1.54		0.84–1.59		0.79–1.53	
Hospital admissions (chronic HBV - infection related)	Patients with events	181	11.0%	36	2.2%	63	3.8%	92	5.6%	113	6.9%
	Count of events	373		53		95		149		205	
	Rate of events PPY	0.23		0.07		0.07		0.06		0.05	
	95%CI of rate	0.19–0.27		0.05–0.10		0.05–0.09		0.04–0.08		0.04–0.06	
Outpatient/GP (all-cause)	Patients with events	1615	98.5%	1583	96.6%	1595	97.3%	1596	97.4%	1599	97.6%
	Count of events	16,792		7620		13,582		22,668		37,534	
	Rate of events PPY	10.33		9.97		9.38		8.93		8.56	
	95%CI of rate	9.71–10.98		9.43–10.54		8.91–9.88		8.49–9.39		8.18–8.97	
ER visits (all-cause)	Patients with events	443	27.0%	226	13.8%	355	21.7%	493	30.1%	631	38.5%
	Count of events	989		372		678		1203		2063	
	Rate of events PPY	0.61		0.49		0.47		0.47		0.47	
	95%CI of rate	0.53–0.69		0.42–0.57		0.41–0.54		0.42–0.54		0.42–0.52	
Hospital days (all-cause)	Patients with events	400	24.4%	179	10.9%	261	15.9%	359	21.9%	474	28.9%
	Count of events	11,379		2927		4414		7148		10,405	
	Rate of events PPY	7		3.83		3.05		2.81		2.37	
	95%CI of rate	5.96–8.21		2.10–6.98		1.75–5.32		1.76–4.50		1.54–3.65	

Hospital days (chronic HBV infection-related)	Patients with events	173	10.6%	28	1.7%	53	3.2%	78	4.8%	98	6.0%
	Count of events	3754		212		467		710		954	
	Rate of events PPY	2.31		0.28		0.32		0.28		0.22	
	95%CI of rate	1.84–2.90		0.16–0.47		0.20–0.51		0.18–0.43		0.14–0.33	
IFN prescription	Patients with events	≤4	≤0.2%	≤4	≤0.2%	≤4	≤0.2%	≤4	≤0.2%	≤4	≤0.2%
	Count of events	13		≤4		≤4		≤4		≤4	
	Rate of events PPY	0.01		≤0.01		≤0.00		≤0.00		≤0.00	
	95%CI of rate	0.00–0.04		NA		NA		NA		NA	
NA prescription	Patients with events	539	32.9%	433	26.4%	442	27.0%	449	27.4%	463	28.3%
	Count of events	8419		2101		3516		5681		9022	
	Rate of events PPY	5.18		2.75		2.43		2.24		2.06	
	95%CI of rate	4.53–5.92		2.38–3.18		2.10–2.80		1.95–2.57		1.81–2.34	
All prescription	Patients with events	1316	80.3%	1191	72.7%	1273	77.7%	1335	81.5%	1381	84.3%
	Count of events	99,774		32,119		54,994		91,709		147,113	
	Rate of events PPY	61.35		42.02		37.99		36.11		33.56	
	95%CI of rate	54.77–68.73		36.72–48.09		33.51–43.06		32.05–40.69		30.01–37.53	

ER, emergency room; GP, general practitioner; HBV, hepatitis B virus; IFN, interferon; NA, nucleos(t)ide analogue.

Supplementary Figure A1. Durability of HBsAg loss

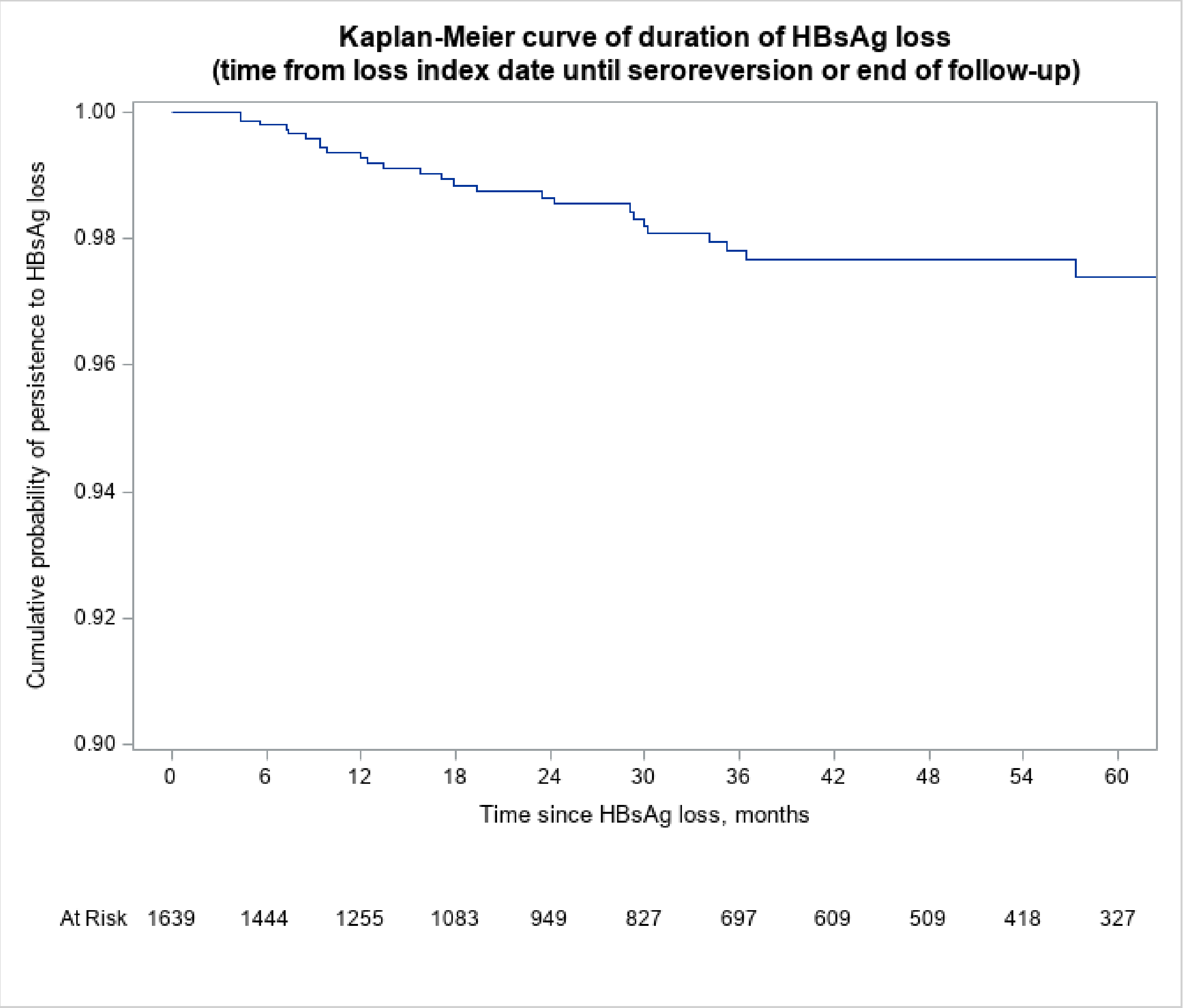

HBsAg, hepatitis B surface antigen.

Supplementary Figure A2. Durability of HBsAg loss from loss index date until seroreversion or end of follow-up stratified by sex, HBeAg status, history of cirrhosis, and treatment

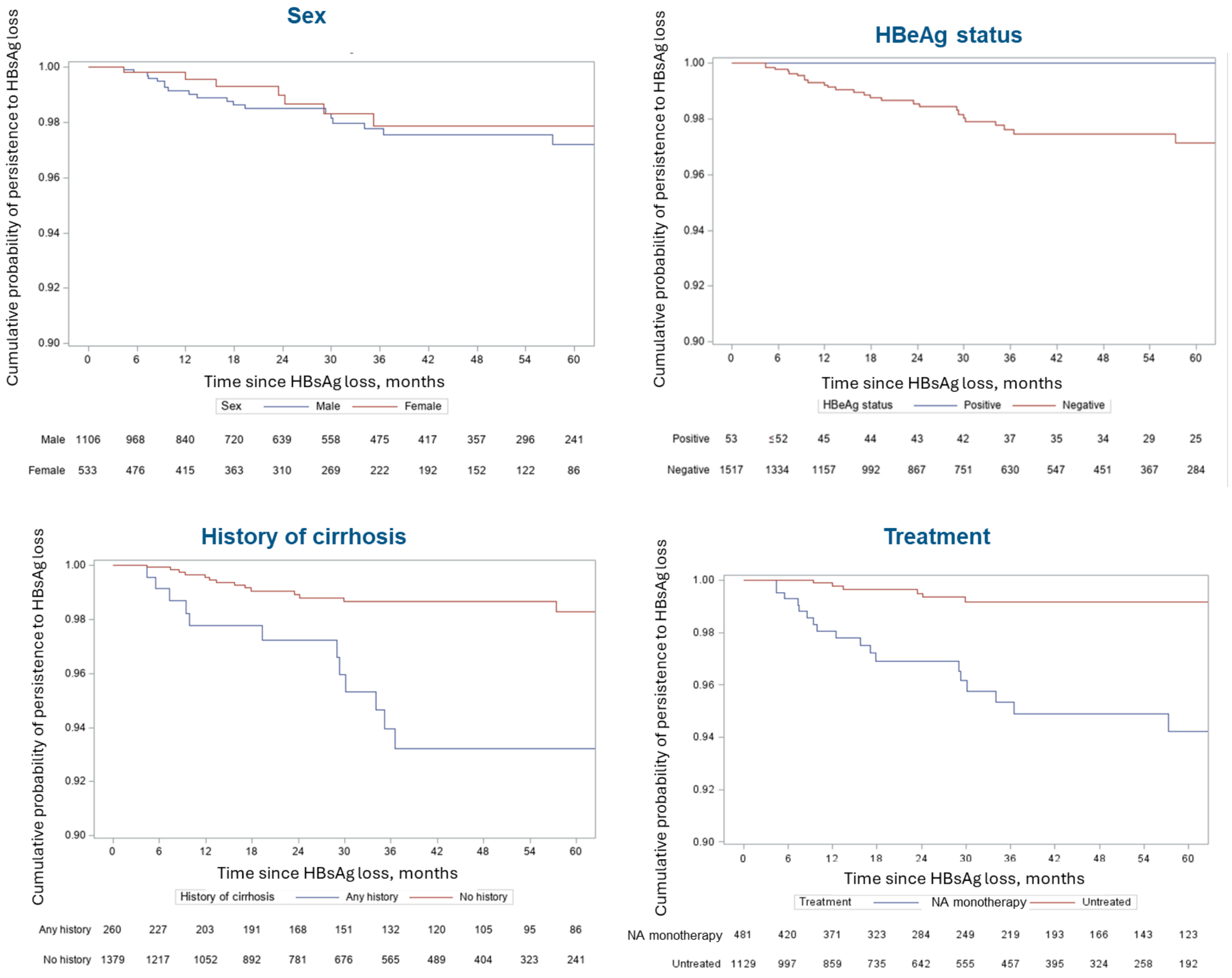

HBeAg, hepatitis B e antigen; HBsAg, hepatitis B surface antigen.

Supplementary Figure A3. Cumulative clinical benefit of HBsAg loss over time on the risk of HCC

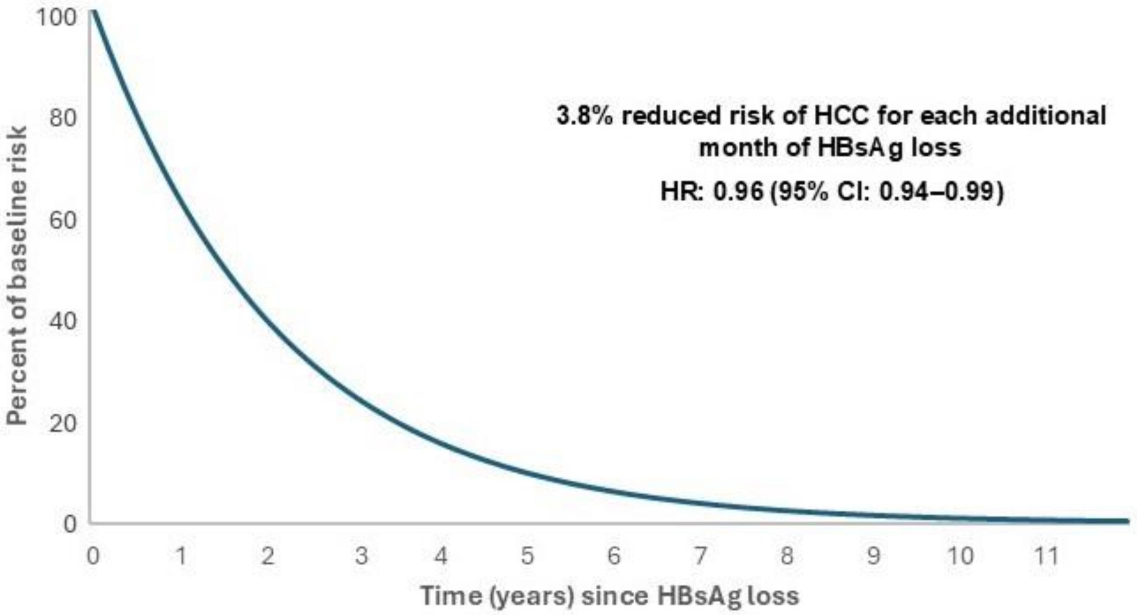

Supplement: Extended PDF [file mmc2.pdf]
